# PredictMed-CDSS: Artificial Intelligence-Based Decision Support System Predicting the Probability to Develop Neuromuscular Hip Dysplasia

**DOI:** 10.3390/bioengineering12080846

**Published:** 2025-08-06

**Authors:** Carlo M. Bertoncelli, Federico Solla, Michal Latalski, Sikha Bagui, Subhash C. Bagui, Stefania Costantini, Domenico Bertoncelli

**Affiliations:** 1 Department of Computer Science, Hal Marcus College of Science & Engineering, University of West Florida, Pensacola, FL 32514, USA; bagui@uwf.edu (S.B.); sbagui@uwf.edu (S.C.B.); domenico.bertoncelli@gmail.com (D.B.); 2 Department of Pediatric Orthopedic Surgery, Pediatric University Hospital Lenval, 06000 Nice, France; federico.solla@hpu.lenval.com; 3 Children Orthopedic Department, Children University Hospital of Lublin, 20-093 Lublin, Poland; michall1@o2.pl; 4 Department of Information Engineering, Computer Science and Mathematics, University of L’Aquila, 67100 L’Aquila, Italy; stefania.costantini@univaq.it

**Keywords:** cerebral palsy (CP), neuromuscular hip dysplasia (NHD), ensemble of machine learning (ML) algorithms, neural network (NN), Support Vector Machine (SVM), logistic regression (LR), Clinical Decision Support System (CDSS)

## Abstract

Neuromuscular hip dysplasia (NHD) is a common deformity in children with cerebral palsy (CP). Although some predictive factors of NHD are known, the prediction of NHD is in its infancy. We present a Clinical Decision Support System (CDSS) designed to calculate the probability of developing NHD in children with CP. The system utilizes an ensemble of three machine learning (ML) algorithms: Neural Network (NN), Support Vector Machine (SVM), and Logistic Regression (LR). The development and evaluation of the CDSS followed the DECIDE-AI guidelines for AI-driven clinical decision support tools. The ensemble was trained on a data series from 182 subjects. Inclusion criteria were age between 12 and 18 years and diagnosis of CP from two specialized units. Clinical and functional data were collected prospectively between 2005 and 2023, and then analyzed in a cross-sectional study. Accuracy and area under the receiver operating characteristic (AUROC) were calculated for each method. Best logistic regression scores highlighted history of previous orthopedic surgery (*p* = 0.001), poor motor function (*p* = 0.004), truncal tone disorder (*p* = 0.008), scoliosis (*p* = 0.031), number of affected limbs (*p* = 0.05), and epilepsy (*p* = 0.05) as predictors of NHD. Both accuracy and AUROC were highest for NN, 83.7% and 0.92, respectively. The novelty of this study lies in the development of an efficient Clinical Decision Support System (CDSS) prototype, specifically designed to predict future outcomes of neuromuscular hip dysplasia (NHD) in patients with cerebral palsy (CP) using clinical data. The proposed system, PredictMed-CDSS, demonstrated strong predictive performance for estimating the probability of NHD development in children with CP, with the highest accuracy achieved using neural networks (NN). PredictMed-CDSS has the potential to assist clinicians in anticipating the need for early interventions and preventive strategies in the management of NHD among CP patients.

## 1. Introduction

**Figure 1:**
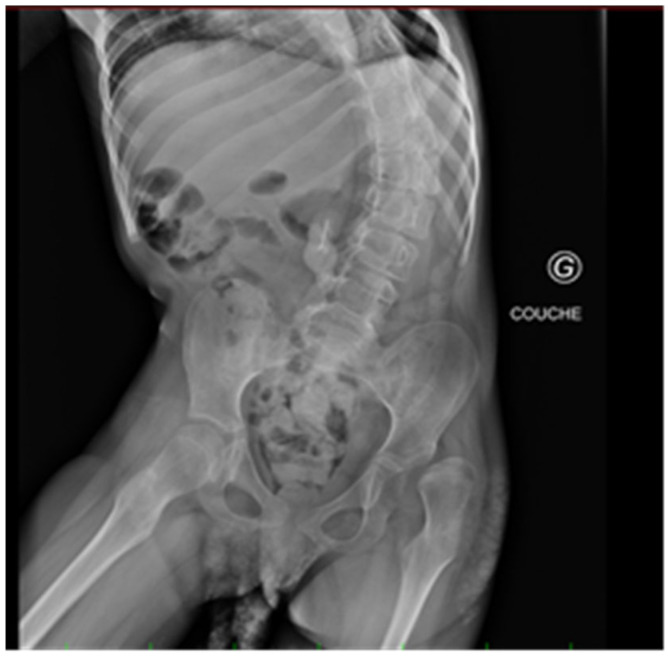
Pelvic X-ray: subluxation of the left hip and coxa valga with thoraco-lumbar scoliosis.

Hip dysplasia and dislocation are prevalent and result in serious issues in children with cerebral palsy (CP) (Figure 1). The risk of neuromuscular hip dysplasia (NHD) is particularly elevated in patients with the most severe forms of CP, especially in those who are non-ambulatory. Dislocation may lead to pain and severe problems with verticalization, sitting balance, perineal nursing care, and decubitus ulceration. Therefore, special screening programs have been developed to diagnose and treat hip displacement early to prevent complete dislocation [1,2,3].

Currently, the Gross Motor Function Classification System (GMFCS) is the only confirmed strong indicator of risk for NHD [3,4,5]. However, other factors could be influential, and there is still a need for further research in this field since the rate of deterioration of the hips has not been established [1]. At present, there are no efficient tools capable of predicting NHD in children with CP.

Artificial intelligence (AI), along with its foundational techniques in machine learning (ML) and neural networks, has advanced significantly in recent years. These developments have enabled computers to outperform human capabilities in areas once considered exclusively human, such as neurology and orthopedics. Our study focuses on AI-driven diagnostics, aiming to familiarize clinicians and researchers with the potential of machine intelligence in neurological practice and clinical research.

A Clinical Decision Support System (CDSS) is a computer program designed to aid in diagnosing and treating diseases. The integration of mathematical sciences, engineering principles, and computer technology in managing various illnesses has significantly increased. Specialists often diagnose or classify diseases for individual patients or groups using a set of rules. The CDSS collects these rules and uses an inference engine using ML algorithms to evaluate the rule base for a given set of inputs. This method allows for some imprecision in user inputs and rule-based specifications, mimicking the cognitive decision-making ability of specialists and enabling clinicians to achieve more accurate diagnoses. Early diagnosis and detection of associated features are crucial for children with CP and typically require the expertise of an experienced specialist. Therefore, such an expert system can be extremely valuable in areas where specialist services are unavailable. Its use is recommended to shorten the delay and improve the accuracy of diagnosis in patients with suspected NHD [6]. Predictive studies exist on radiological images or on mixed clinical and radiological data [6,7,8] to better help doctors to plan medical interventions to prevent NHD, but predictive models on clinical data alone are necessary in an effort to limit healthcare costs and irradiation

In previous studies, a predictive model for comorbidities in subjects with CP named PredictMed [5,6] was developed to identify risk factors of NHD using Electronic Health Records (EHR) and logistic regression algorithms. EHR data are incredibly valuable for identifying health outcomes. They encompass a wealth of clinical information, such as laboratory test results, vital signs, discharge summaries, progress notes, and radiologic and pathologic images and reports, among other details. Although the previous study allowed recognition of strong predictors, its performance was suboptimal, with low sensitivity [5]. Therefore, we implemented a novel CDSS prototype, called PredictMed-CDSS, to calculate the probability of developing NHD from clinical (non-image) data in new incoming children with CP. For this, we implemented a ML model based on an ensemble of three ML classifiers: Neural Networks (NN), Support Vector Machines (SVM), and Logistic Regression (LR) [9,10,11,12].

From a clinical point of view, the availability of a robust CDSS providing the probability of developing NHD in children with CP would allow healthcare providers to better care for patients at risk with increased posture equipment, physiotherapy, X-ray assessment, spasticity treatment, and less-invasive surgery. From a research point of view, PredictMed-CDSS could be helpful in the implementation of future machine-learning models in other neurological and orthopedic fields.

## 2. Materials and Methods

### 2.1. Study Design

This longitudinal, multicenter, and international cohort study was conducted between June 2005 and June 2023. The external validation adhered to the guideline for the early-stage clinical evaluation of decision support systems driven by artificial intelligence: DECIDE-AI [13]. In a double-blind study, we compared two groups of children with CP, motor disorders, and cognitive impairment treated in specialized units. There were no differences from the developmental data in setting, eligibility criteria, outcome, and predictors.

### 2.2. Subjects

The inclusion criteria were as follows: subjects aged between 12 and 18 years at the last follow-up; diagnosed with spastic, dystonic, mixed spastic/dystonic, or hypotonic CP as per the Surveillance of Cerebral Palsy in Europe system [13]; and having a minimum of three years of follow-up (6.4 ± 1.2 years, range 3 to 12). Exclusion criteria included progressive encephalopathy or spinal cord neuropathology, with no missing data.

**Figure 2:**
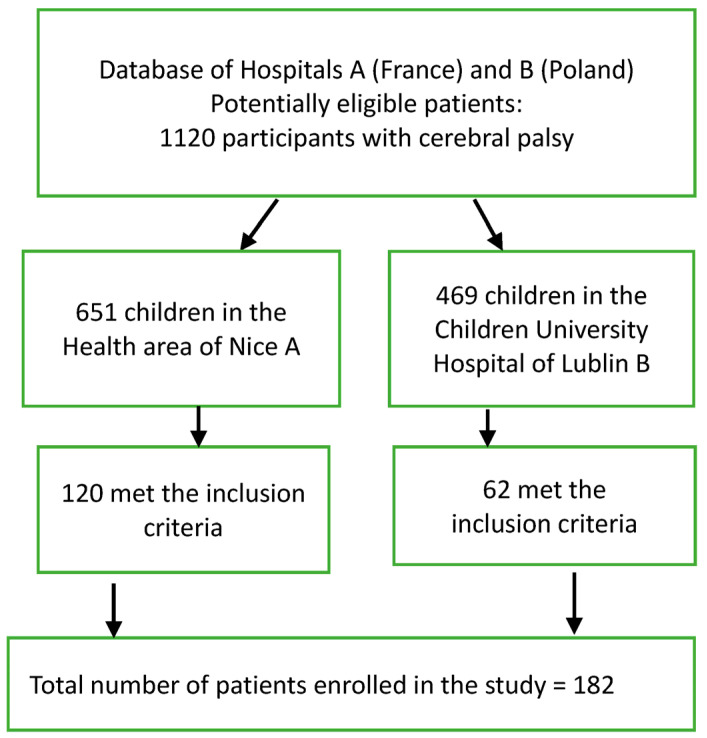
Flow diagram of study participants for analysis.

Of 651 children with a diagnosis of CP in the Nice region (France), 120 (74 males, age 16.5 ± 1.8 years) from the Pediatric University Hospital Lenval (named “A”) met the inclusion criteria. The database of the Children’s University Hospital of Lublin, Poland, (named “B”) included 469 patients with a diagnosis of CP, and 60 (30 males, age 15.9 ± 1.8 years) of these met the inclusion criteria (Figure 2).

### 2.3. Measurements

Data were collected from the EHRs by a multidisciplinary team, including neurologists, pediatricians, physiotherapists, epidemiologists, and orthopedic surgeons. Data on diagnosis, etiology, functional assessments, type of epilepsy, spasticity, hip radiology, and clinical history were gathered between 2005 and 2023. Narrative notes were examined, coded, and anonymously inputted into the electronic database for the neuro-orthopedic condition known as “PredictMed” [5,6].

The etiology of CP was classified as antenatal (cerebral malformation, infection, vascular, or genetic), perinatal (ischemic, infectious, or anoxic), or postnatal (postnatal anoxic/ischemic injury, infectious, cranial trauma, or epilepsy) [14,15].

**Figure 3:**
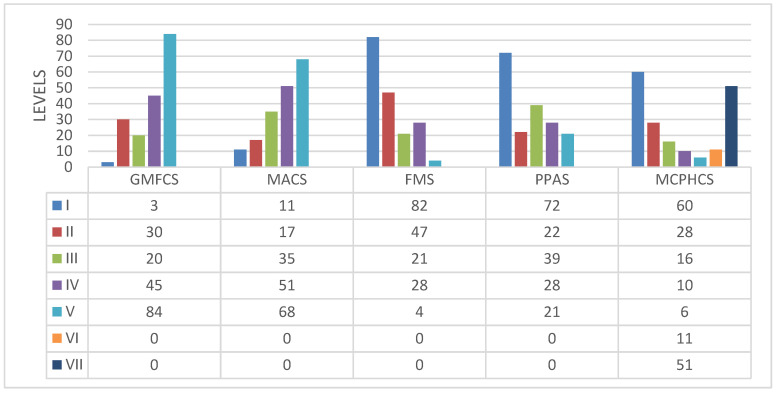
Distribution of patients according to the Gross Motor Function Classification System (GMFCS), Manual Ability Classification System (MACS), Functional Mobility Scale (FMS). Posture and Postural Ability Scale (PPAS), and Melbourne Cerebral Palsy Hip Classification Scale (MCPHCS). Non-applicable (0).

Assessment of motor function was conducted using the Manual Ability Classification System (MACS) and the Gross Motor Function Classification System (GMFCS) [14,15] (Figure 3); both utilize a 5-point classification system where higher scores indicate poorer motor functioning.

Walking capacity was defined as walking independently without help or support, and classified as yes or no.

The tone of trunk muscles was evaluated using the Trunk Impairment Scale (TIS) [5], which includes assessments of static and dynamic sitting balance and categorizes muscle tone as hypotonic, spastic, or normal. Functional abilities were assessed with the Lower Extremity Functional Scale (LEFS), the Posture and Postural Ability Scale (PPAS), and the Functional Mobility Scale (FMS) [16,17,18] (Figure 3). The LEFS scores lower extremity function on an 80-point scale, where higher scores indicate improved function. The PPAS evaluates sitting posture through a 7-level classification system, with higher scores reflecting better postural control. The FMS measures functional mobility and walking capacity through a 6-level classification system, where higher scores signify improved motor functioning.

- Scoliosis was identified by a Cobb angle exceeding 10° on a spinal radiograph, with a designation of “severe” scoliosis given when the Cobb angle surpassed 40° or required surgery [5,15] (Table 1).
- Neurologic status was categorized based on the topography of spastic disorder (hemiplegia, diplegia, tri/quadriplegia), the presence of hypertonia in the upper or lower limbs, the presence of dystonia, and the severity of epilepsy.
- Spasticity was measured using the Bohannon and Smith modified Ashworth Scale and the Modified Tardieu Scale [14]. Dystonia is a neurological hyperkinetic movement disorder where continuous or repetitive muscle contractions lead to twisting and repetitive movements or unusual fixed postures. The presence of dystonia was classified as either present or absent through a clinical evaluation [19,20].
- Pediatric neurologists assessed the severity of epilepsy as either “well controlled” or “intractable” based on the guidelines set by the International League Against Epilepsy. Intractable epilepsy is defined as continued seizures despite treatment with a minimum of two antiepileptic medications [6] (Table 1).
- The clinical assessment of the hip primarily focused on internal rotation and hip abduction. The Melbourne Cerebral Palsy Hip Classification Scale (MCPHCS) was used to classify hip morphology. The modified Harris Hip Score (MHHS) was utilized to evaluate hip function, gait, and pain. All patients underwent at least one pelvic X-ray, with the most recent one being reviewed by a pediatric orthopedic surgeon in cases where multiple X-rays were available [1,2,3].

The evaluation of dysplasia was conducted using the Perkins’ line criteria. If the lateral margin of the femoral head was positioned medial to Perkins’ line and the migration percentage (MP) was negative, it was assigned a value of 0%. Conversely, the MP was recorded as 100% if the entire femoral head was lateral to the Perkins line. Based on the migration percentage, the hips were categorized as normal (MP below 33%), subluxation (MP ranging from 33% to 89%), or dislocation (MP equal to or greater than 90%) [1,3].

The type of spasticity (SP), etiology (ET), truncal tone (TT), presence of dystonia (D), and epilepsy (E), MACS score, GMFCS score, walking capacity (W), and sex (SE) were assessed at first assessment; neuromuscular scoliosis (NS) evaluation took place during the final control.

### 2.4. Data Analysis

#### 2.4.1. Univariate Analysis

We used Fisher’s exact tests to assess association between categorical variables , based on the exact probability under null hypothesis of independence. If the *p*-value is less than the chosen significance level (α), the null hypothesis of no association is rejected. We also calculated odds ratio (OR) and the effect size (holding other variables fixed). More specifically, if,

- OR = 1, No association between the variables. The predictor variable does not affect the odds of the outcome.
- OR > 1, Positive association between variables. An increase in the predictor variable is positively associated with higher odds of the outcome.
- OR < 1, Negative association between the variables. An increase in the predictor variable is (negatively) associated with lower odds of the outcome.

That is, if OR = 2, the odds of the outcome are twice as likely for a 1-unit increase in the predictor, and if OR = 0.5 the odds of the outcome are reduced by 50% for a 1-unit increase in the predictor.

#### 2.4.2. The PredictMed-Clinical Decision Support System

PredictMed-CDSS was implemented to estimate the probability for a new incoming subject with CP of developing NHD.

**Figure 4:**
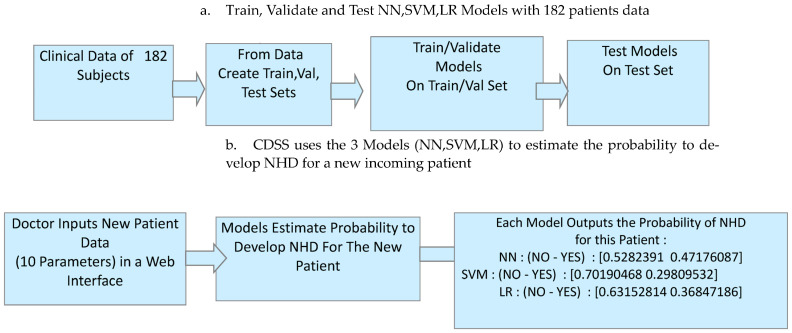
CDSS set-up (**a**) and usage (**b**): (**a**) train, validate, and test three ML models and (**b**) use them to predict hip instability in new incoming patients.

Based on univariate analysis and literature data [1,2,3,4,5,6,7,8], we selected 10 parameters: type of truncal tone (TT), spasticity (SP), epilepsy (E), etiology (ET), sex (SE), presence of dystonia (D), neuromuscular scoliosis (NS), history of orthopedic surgery (SU), and GMFCS and MACS scores. Thus, each trained classifier estimates a probability of developing NHD for that patient (Figure 4).

In addition to the probability estimation from each of the three classifiers, the CDSS provides the predictive performance metrics of each classifier to give medical personnel with an estimation of the reliability of the probability estimation itself. The classifiers’ models are then used to estimate the probability of developing future NHD in a new patient, after taking in the new patient data parameter input: TT, SP, E, ET, SE, D, NS, SU, GMFCS, MACS

##### System Architecture

**Figure 5:**
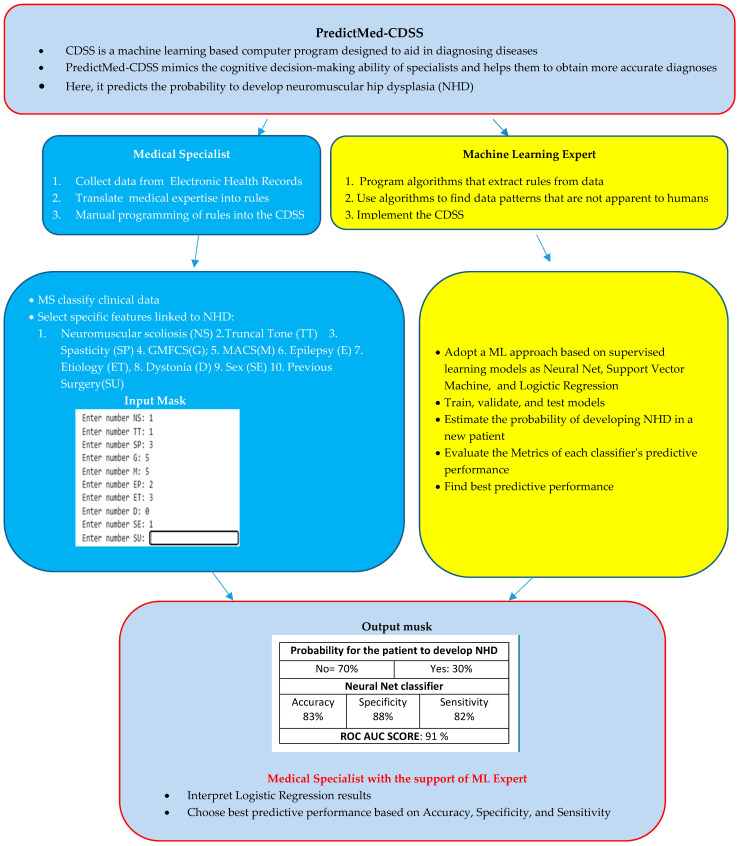
PredictMed-CDSS plot.

In this study, we adopted a ML algorithm based on three supervised learning models (NN, SVM, LR) for calculating the probability of NHD in 182 subjects with CP using their statistical non-image clinical data. We identified predictors after a feature reduction previously described in the Data Source section. The block diagram structure of the PredictMed-CDSS is described in Figure 4 and Figure 5.

The dataset was relatively small and imbalanced, with 121 non-NHD and 61 NHD cases. To improve model generalization under these constraints, we employed an ensemble of three simple machine learning models (NN, SVM, and LR). Clinical data were randomly split into a training/validation set (145 patients) and a test set (37 patients). These data were input into three classifiers—Neural Networks (NN), Support Vector Machines (SVM), and Logistic Regression (LR)—which learned from the training set. The predictive performance of each classifier was evaluated on the test set using standard metrics, including accuracy, sensitivity, specificity [21], and the area under the receiver operating characteristic curve (AUROC) [22].

To mitigate overfitting, we applied several regularization techniques. For the NN model, we used early stopping [23], which halts training before overfitting occurs, as well as dropout and weight regularization [24] to enhance generalization. The SVM model incorporated regularization through the hyperparameters *C* and *gamma*, optimized using GridSearchCV with five-fold cross-validation. Similarly, the LR model benefited from built-in regularization via the *C* parameter, also tuned using five-fold cross-validation with GridSearchCV.

Additionally, to further enhance generalization and reduce overfitting, we implemented an ensemble approach [25] combining predictions from all three classifiers (NN, SVM, and LR).

To address class imbalance, we applied a class-weighting scheme across all models, which assigns higher importance to minority class samples. Specifically, we used the compute_class_weight() utility function from the scikit-learn library [26], which calculates appropriate weights based on class distribution in the training set, ensuring balanced penalization during model training.

##### Predictive Metrics

To evaluate the predictive performance of each classifier, we used four commonly defined outcomes: true positives (TPs), true negatives (TNs), false positives (FPs), and false negatives (FNs). TP represents correctly predicted NHD cases, while TN refers to correctly predicted non-NHD cases. FP and FN correspond to incorrectly predicted NHD and non-NHD cases, respectively.

Accuracy (Acc) was calculated as follows: Acc = (TP + TN)/(TP + FN + FP + TN). Although Acc is a widely used metric for evaluating classification models, it can be misleading in imbalanced datasets. In our case, the dataset was imbalanced, with more non-NHD cases (n = 121) than NHD cases (n = 61). To address this issue, we also calculated sensitivity (Sn) and specificity (Sp).

Sensitivity (also known as recall or true positive rate) is the proportion of actual NHD cases correctly identified by the model, as follows: Sn = TP/(TP + FN)

Specificity (true negative rate) is the proportion of non-NHD cases correctly identified, as follows: Sp = TN/(TN + FP)

**Figure 6:**
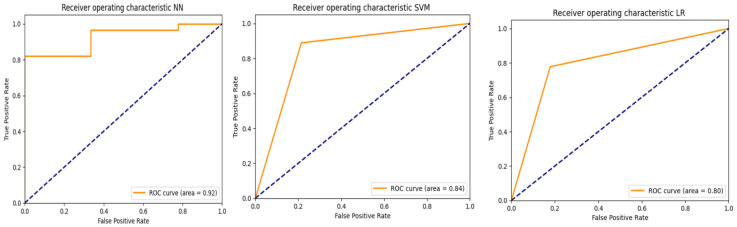
AUROC (Area under receiver operating characteristic). The blue dashed line represents the curve of a random classifier (AUROC = 0.5), serving as a baseline : the further above this line the model's ROC curve lies, the better its ability to discriminate between classes.

The area under receiver operating characteristic (AUROC) [22,27] was also used to evaluate the predictive performance of the three models (NN, SVM, LR), as shown in Figure 6.

The AUROC measures the ability of a model to distinguish between positive and negative classes. An AUROC value of 1.0 indicates perfect classification performance, while a value of 0.5 or below suggests the model performs no better than random guessing—or worse. ROC curves were generated based on the predictions obtained from the test set.

To address the issue of class imbalance during model training, we employed the compute_class_weight() utility function from the scikit-learn library [26]. This function analyzes the distribution of class labels and assigns appropriate weights, ensuring that underrepresented classes are given proportionally greater importance, thus helping to balance the impact of each class during training.

Pre-processing steps included feature scaling to ensure faster and more stable model training, balanced learning across features, and improved overall model performance. Prior to being input into the machine learning models, the data were standardized using the StandardScaler() function from the Python scikit-learn library, version 1.6.1 [26].

##### Neural Network (NN) Model Architecture

The NN model was implemented using the Keras, version 3.8.0 [28] and TensorFlow, version 2.18.0 [29] libraries. A simple feed-forward neural network was trained using the standard backpropagation algorithm. Multiple hyperparameters were tuned, including the number of hidden layers, the number of neurons per layer, activation functions [30], optimization method, and regularization techniques [24], including dropout [31].

The optimal architecture consisted of four hidden layers with 128, 64, 32, and 2 neurons, respectively. Rectified linear unit (ReLU) activation functions were used in all hidden layers, while the output layer used a softmax activation function to provide class probabilities. To prevent overfitting, dropout layers with a dropout rate of 0.5 were inserted after each hidden layer, and L2 regularization was applied.

**Figure 7:**
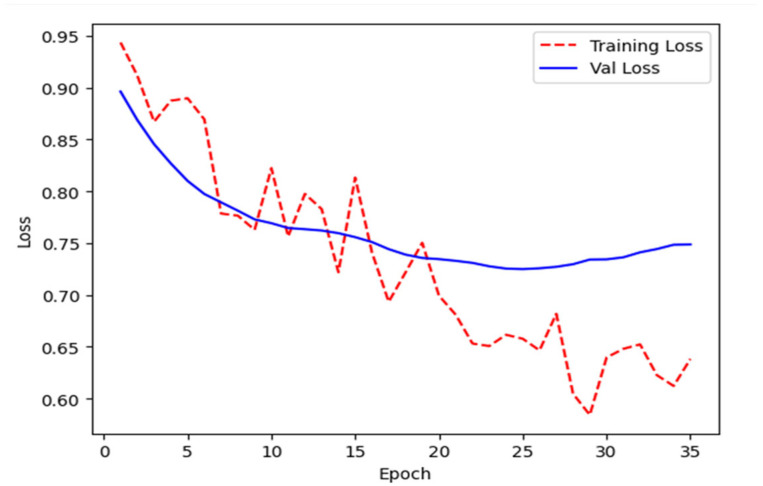
Training loss vs. validation loss for early stopping monitoring.

NN was trained using validation data [10] and incorporated early stopping [23] to avoid overfitting. Training and validation loss curves (Figure 7) were plotted to monitor the model’s learning behavior, confirm the correct implementation of early stopping, and ensure balanced performance—avoiding both underfitting and overfitting. Early stopping automatically halted training when validation loss began to diverge from training loss, signaling the onset of overfitting.

The Adam optimizer [32] was used due to its robustness and empirical effectiveness across a wide range of hyperparameters. The loss function was sparse categorical cross-entropy [33], suitable for multiclass classification tasks, such as predicting the presence or absence of NHD. Given the class imbalance in the dataset, we applied a class weight scheme [34], which assigned greater importance to the minority class (NHD), thereby improving the model’s sensitivity to underrepresented cases.

The training and validation datasets were derived from 145 patients (out of 182 total), and were used to monitor loss during training.

To further enhance training efficiency and convergence, a learning rate schedule was implemented using TensorFlow’s tf.keras.optimizers.schedules.ExponentialDecay function [35]. This allowed the learning rate to start high for faster initial convergence and then gradually decrease during training to fine-tune model weights. This dynamic approach outperformed the use of a fixed learning rate.

Finally, the probability of a CP patient developing NHD was computed using the model.predict() function in Keras [35], providing the basis for risk estimation in our PredictMed-CDSS system.

##### Support Vector Machine (SVM) Model Architecture

The Support Vector Machine (SVM) model was implemented using the scikit-learn library [26]. To optimize model performance, the GridSearchCV function was used to perform hyperparameter tuning with 5-fold cross-validation [36]. This process identified the optimal values for key parameters—including C, tol, and kernel—by finding the best trade-off between underfitting and overfitting [37].

Early stopping was employed using a tolerance (tol) value of 0.1, which ensures the algorithm stops only after sufficient convergence. This helps prevent premature termination that could result in an undertrained model—particularly relevant when working with small datasets. Moderate regularization was applied with C = 1, a value chosen to balance model complexity and generalization. In SVM, the C parameter controls the trade-off between achieving a low training error and maintaining a wider margin to reduce overfitting. A smaller C encourages a simpler, more generalizable model, while a larger C favors fitting the training data more closely. The selected value (C = 1) reflects a balanced compromise between these extremes [11,38,39].

To handle class imbalance, class weights were automatically adjusted using the class_weight=‘balanced’ option in the scikit-learn SVM implementation. This approach ensures that the minority class (NHD) receives proportional influence during training.

An RBF (radial basis function) kernel with degree = 3 was selected to capture nonlinear relationships in the data, making the model more flexible in handling complex clinical patterns.

Finally, the probability of a CP patient developing NHD was estimated using the model.predict() function from the scikit-learn SVM library [26], providing individual risk scores for integration into the PredictMed-CDSS.

##### Logistic Regression (LR) Model Architecture

The Logistic Regression (LR) model was implemented using the scikit-learn library functions for logistic regression [26]. The model was optimized using GridSearchCV with 5-fold cross-validation to effectively handle the small and imbalanced dataset. Class weights were automatically balanced using the class_weight=‘balanced’ option, which ensures that the minority class (NHD) is appropriately weighted, thereby improving predictive performance on underrepresented cases [40].

The lbfgs solver was selected as the optimization algorithm, as it is efficient and well suited for small- to medium-sized datasets, allowing for rapid convergence even with limited training data. The regularization parameter was set to C = 0.0001, which increases the regularization strength. This helps mitigate overfitting by penalizing overly complex models, thus promoting better generalization.

To estimate the probability that a subject with cerebral palsy (CP) will develop NHD, the model.predict() function from the scikit-learn LogisticRegression module was used [26].

### 2.5. Institutional Review Board Statement

All practices were conducted in accordance with University Hospital ethical standards and the 1964 Declaration of Helsinki and its subsequent amendments. Data were anonymized and analyzed according to the requirements of Reference Method 003, numbered “2017728 v 0.” Ethics committee approval and informed consent were recorded as “2017728 v 0-MR003 (Reference Method 003) 27 March 2017.” All participants and parents consented to participate.

## 3. Results

Table 1 displays the clinical presentation of participants based on their NHD status and neurological statuses. At the most recent follow-up (6.6 ± 1.4 years after baseline, ranging from 3 to 12 years), 61 individuals (34%) were identified as having NHD, with 36 of them (62%) experiencing dislocated hips.

In terms of hip range of motion, 30% (n = 54) of patients exhibited uni- or bilateral internal rotation exceeding 40°, while 56% (n = 102) had limited hip abduction of less than 20°. As for radiological hip morphology, 66% (n = 121) had normal or near normal hips, 8% (n = 14) had dysplastic hips, 4% (n = 7) had dysplasia with mild subluxation, 2% (n = 4) had moderate to severe subluxation, and 20% (n = 36) had dislocated hips.

Botulinum toxin was injected in the adductor muscles of 64 patients, with seven of them also receiving injections in the hip flexors.

In total, 45 patients underwent hip surgery, with 35 of them having a femoral osteotomy, and 16 of these also undergoing an iliac osteotomy. Moreover, 11 patients underwent proximal femoral resection, 8 patients had a bilateral procedure, and 41 underwent multiple tenotomies.

### 3.1. Statistical Analysis

Fisher’s exact test indicated (Table 2) that factors significantly associated with NHD were spasticity, scoliosis, truncal tone disorders, poor manual skills and gross motor function, and disability of standing position and independent walking.

The multivariate logistic regression model (Table 3) revealed that the best predictors of NHD were the following:

- Previous history of orthopedic surgery (*p* = 0.0017);
- Truncal tone disorder (*p* = 0.0049);
- Poor motor function (*p* = 0.063).

**Logistic Regression.** The increasing SU, TT, and MACS scores are associated with neuromuscular hip dysplasia. More precisely, this means, e.g., that for every unit increase in TT, the log odds = ln(p/1 − p) increases 2.4307 times (where p = probability to have neuromuscular hip dysplasia). The “Prob(>|z|)” column indicates the significant strength of the respective parameter in terms of the *p*-value as the presence of neuromuscular hip dysplasia. This means that the significance of SU, MACS, and TT in predicting the presence of neuromuscular hip dysplasia is very probable, with a *p*-value < 0.06.

### 3.2. PredictMed-CDSS Performance Metrics

The predictive performance on the test set is presented in Table 4. Overall accuracy was between 0.81 and 0.84; AUROC was between 0.8 and 0.92. The highest scores were obtained with NN.

Table 5 presents the input and output interface of the Clinical Decision Support System (CDSS), which is accessible via a simple web link. Table 5 also provides a screenshot of the CDSS web interface. In this interface, clinical data for a new patient (10 parameters) are entered through a Google Drive-based web form. The CDSS then outputs the estimated probability of that patient developing NHD, as predicted independently by each of the three models (NN, SVM, and LR).

In addition to the probability estimates, the CDSS displays the performance metrics—accuracy, sensitivity, and specificity—for each model. These metrics, obtained during the training and testing phases, are shown alongside the predictions to give healthcare professionals a measure of the reliability and expected performance of each model for the specific patient being evaluated.

## 4. Discussion

We developed PredictMed-CDSS, a first prototype of a Clinical Decision Support System based on AI and machine learning that can predict the probability of developing NHD in subjects with CP from non-radiological data. The predictMed-CDSS prototype interface, as described in Table 5, is extremely user-friendly: after accessing a web link, the doctor inputs a series of patient parameters and the CDSS calculates the probability of developing a specific outcome (NHD).

A comparison with similar studies shows that the present results concern a larger sample; moreover, the performance was higher than previous literature with only clinical data (Table 6).

This CDSS prototype has been developed for the prediction of NHD, but it is perfectly adaptable to the prediction of different types of other CP outcomes (depending on the available datasets to be used for training the ensemble of classifiers).

AI models must prioritize diverse and representative datasets to reduce inequalities and ensure diagnostic accuracy for various populations. For this, we implemented a model using two samples of subjects with CP from different countries.

In a constantly evolving technological landscape, CDSS has become an essential tool for improving patient care. CDSSs offer healthcare professionals new insights to improve diagnostic accuracy, therapeutic planning, and treatment personalization. Furthermore, they offer cost-effective options to complement conventional screening in secondary prevention. The use of CDSS to predict medical outcomes for various types of pathologies are already present in literature [4,9,13]. For instance, CDSS improved experts’ perceived ability to accurately plan pancreatic cancer resection [41]. Moreover, a CDSS was developed to help nurses managing delirium in intensive care units and was perceived as efficient and easy to use. Furthermore, the use of CDSS allowed improvements in the completeness and agreement of paper-based antenatal records in Nepal. AI can also become a relevant tool in the assessment of leukemia cases and potentially accelerate the screening of this condition [25]. In another field, Devnath et al. [42] proposed three ensemble-learning techniques (simple averaging, multiweighted averaging, and majority voting) for the automatic detection of pneumoconiosis in coal worker’s chest X-ray radiography. Integrating image and clinical image data, Yuan et al. [43] predicted the onset of future cancer when the lungs are “visually normal” on computed tomography. Interestingly, they used a machine learning model (linear discriminate analysis, LDA) that could be employed for feature reduction in the case of high dimensionality of the datasets. Devnath et al. [37,42] proposed three ensemble learning techniques (simple averaging, multiweighted averaging, and majority voting (MVOT)) for the automatic detection of pneumoconiosis in coal worker’s chest X-ray radiography. Even if these studies analyze different data (image data) from ours (non-image data), some of the techniques that were employed (like majority voting) could be interesting for our next research steps. Further, we do not exclude the possibility of using datasets integrating image and non-image data, like Yuan et al. [43] did to predict who will develop future cancer when the lungs are “visually normal” on CT (computed tomography) scans. Another interesting point of this study could be its use of a machine learning model (linear discriminate analysis, LDA) that, in our case, could be employed for feature reduction, in the case of high dimensionality of the datasets to be employed in our next research steps.

In the field of CP and neurological research, ML has been employed in CDSS to predict early insurgency of CP, neurological disorders, rehabilitation outcomes, or to investigate CP patients’ data [27,44,45]. The novelty of our study resides in the development of a CDSS prototype to be specifically used to predict comorbidities in subjects with CP. For this purpose, we leveraged previous research [5,6,14,15,19,46,47] to predict with ML the CP patient outcomes like NHD, need for gastrostomy or neurotoxin treatment, and scoliosis. This study is the first published concerning the development of an ML-based CDSS for the prediction of a specific CP outcome (NHD).

In this study, we also confirmed previous results about poor motor function and the presence of truncal tone disorder, scoliosis, and spasticity, as predictors of NHD in children with CP [6]. Moreover, we highlighted the influence of previous orthopedic surgery on future NHD onset. This is probably not a causal relationship, but rather a consequence of the same primary neuromuscular disorder: generalized spasticity, weakness, and muscular imbalance initially lead to vicious positions, then to multisite muscular retractions and osteoarticular growth disorders. Therefore, the need for orthopedic surgery, regardless of the site, is likely to be associated with musculoskeletal disorders on the hip.

Apart from a low score of GMFCS [3,4,5,40,48], the other conditions were never reported. Comprehensibly, the present study has shown that children with CP with spasticity have the highest risk of NHD (OR > 29); indeed, it is well known that spasticity is a prime movement of orthopedic deformities, but this study confirmed this hypothesis concerning NHD [1,2]. The presence of scoliosis (OR > 4) and truncal tone disorders (OR > 3) were also associated with the development of NHD; the association of pelvic obliquity, scoliosis, and NHD is a common concept [3], whose evidence is highlighted. A reciprocal influence is probable in this context: truncal tone disorders scoliosis can promote the onset of scoliosis and pelvic obliquity; pelvic obliquity can promote the onset of scoliosis and/or hip dislocation; and hip dislocation can increase pelvic obliquity. Poor gross motor function (OR > 8) and manual abilities (OR > 8) were also associated with the development of NHD compared with a similar group that lacked these problems. As expected, good standing position and independent walking are protectors from NHD. These findings follow the concept that poor gross motor function increases the risk of NDH. The similar results from the two centers and in the totality of patients confirm the validity of the prediction model, which can also be applied to other fields of medical research. In this regard, PredictMed-CDSS aimed to identify the phenotypes of patients at risk for developing NHD. Traditionally, finding phenotypic patterns in biomedical data has been undertaken by addressing one specific question at a time using supervised learning [6], in which a computational algorithm searches for patterns among input variables (or features) that model a specific outcome variable.

To summarize, in this approach, PredictMed-CDSS would assist healthcare providers in answering a specific question: What is the risk of developing NHD for this patient? The availability of a robust predictive algorithm would allow for understanding and managing risk factors for NHD, to facilitate early consultation with a qualified orthopedic surgeon, and to improve the overall quality of care for patients and families.

Regarding the benefit of this research, predicting future clinical outcomes will allow medical personnel to anticipate treatment, surgical interventions, and physical therapies. Timely intervention would limit musculoskeletal deformations and decay of hip functions, significantly reducing costs and improving the lifestyle of the patient and their families. In the presence of one or more predictors, frequent clinical evaluation is suggested, with particular emphasis on abduction, internal rotation, and pain. The frequency of pelvic X-rays should be increased, particularly in cases of clinical deterioration (i.e., pain and decreased abduction) [46,49]. Furthermore, in the presence of a high risk of NHD, parents should be informed about the need for increased screening, preventive measures, and, potentially, hip surgery. The following interventions and treatments are suggested, from the simplest and conservative to the most complex and invasive:

1. Physical therapy: Use targeted exercises to improve mobility. For instance, increased conservative measures such as abduction posture and exercises should also be proposed. Stretching exercises can help to maintain muscle flexibility and reduce stiffness. This therapy is important at all ages and its frequency can be modulated according to the risk of developing NHD.
2. Orthotic devices: Use braces or supports to stabilize the hip joint and prevent further dislocation, with particular emphasis on adduction, especially in case of adductor spasticity or pelvic obliquity.
3. Botulinum toxin injections: Administered to manage spasticity to target specific muscles causing abnormal tone and reduce involuntary contractions, as adductors, but also internal or external rotators and/or flexors.
4. Early and less-invasive surgical interventions, as abductor muscle tenotomy, obturator nerve neurotomy, and hemi-epiphysiodesis of femoral neck to decrease coxa valga. These are often proposed during multisite surgeries to improve muscular balance and global posture, with the aim of avoiding less conservative surgery.
5. Neurosurgical treatment of generalized spasticity: Intrathecal baclofen therapy (ITB) delivers baclofen directly to the spinal fluid, especially in quadriplegic adolescents, or selective dorsal rhizotomy, especially in diplegic younger children. Although they are not specific for hip dysplasia, releasing spasticity could decrease its risk.
6. Osteotomies: Apply femoral varus and/or derotation osteotomy and iliac osteotomy to correct hip alignment and alleviate pain in the case of dysplasia and stiffness [46,49].
7. Non-conservative surgery: Total hip replacement or proximal femoral resection (head and neck) to manage pain, especially in long lasting subluxation/dislocation and damaged cephalic cartilage.

## 5. Limitations, Conclusions, and Future Work

We developed and validated PredictMed-CDSS, a prediction model-based CDSS of NHD. The main limitations of this study include the relatively small sample size and the retrospective nature of the data analysis. These factors may restrict the generalizability of the model and increase the risk of overfitting, but several machine learning techniques were employed to mitigate these issues. However, we acknowledge that there remains significant room for improvement, in terms of both dataset size and the predictive performance of the algorithms. To enhance the stability and reliability of the model, it is crucial to increase the number of patient records while maintaining a manageable number of independent variables (ideally fewer than 15). To address these limitations, we plan to retrain, validate, and test our CDSS models on a larger, nationally representative database. This will allow for a more robust evaluation and optimization of the models’ predictive performance. Additionally, we aim to improve the predictive algorithm of the CDSS. The current ensemble—based on relatively simple machine learning models (NN, SVM, and LR)—is a foundational implementation, and there is considerable potential for enhancement. Refining the ensemble strategy and incorporating more advanced algorithms should contribute to improved predictive accuracy in estimating the probability of developing NHD in patients with cerebral palsy.

In addition to the GMFCS, PredictMed-CDSS has identified a history of previous orthopedic surgery, truncal tone disorder, scoliosis, and spasticity as risk factors for NHD. In individuals with CP and the mentioned predictors of NHD, it is recommended to increase the frequency of clinical hip examinations and hip X-rays to easily identify potential candidates for treatment and appropriate preventative rehabilitation interventions can be adapted. The best predictive model was that based on the neural network. This results in a user-friendly prototype of medical CDSS that estimates the probability for a single CP patient of developing NHD by giving as input to the CDSS 10 clinical parameters, with stronger performance than previous models. Future research will study how to implement a new and better performing ensemble of ML models.

## Figures and Tables

**Table 1:** Clinical presentation based on the presence or absence of neuromuscular hip dysplasia.

	Pediatric Hospital A		Children Hospital B	Multicenter	
Patients Profile	Neuromuscular Hip Dysplasia		Neuromuscular Hip Dysplasia	Total (%)	
	Yes (%)	No (%)	Total (%)	Yes (%)	No (%)
Patients n. (%)	33 (27)	87 (73)	120 (100)	28 (45)	34 (65)
Male	22 (30)	52 (70)	74 (100)	12 (39)	18 (61)
Female	11 (24)	35 (76)	46 (100)	16 (50)	16 (50)
Average age (mean, SD)	16.3 (1.8)	16.7 (1.8)	16.5 (1.8)	15.8 (1.8)	16.0 (1.8)
Antenatal causes	27 (36)	48 (64)	75 (100)	9 (33)	17 (67)
Perinatal causes	14 (45)	17 (55)	31 (100)	18 (54)	15 (46)
Postnatal causes	3 (21)	11 (79)	14 (100)	1 (33)	2 (67)
Spasticity n. (%)	29 (32)	62 (68)	91 (100)	28 (45)	34 (65)
Hemiplegia	2 (20)	8 (80)	10 (100)	1 (9)	10 (91)
Diplegia	1 (5)	18 (95)	19 (100)	9 (30)	21 (70)
Tri/quadriplegia	26 (42)	36 (58)	62 (100)	18 (89)	3 (11)
Dystonia n. (%)	3 (20)	11 (80)	14 (100)	8 (58)	6 (42)
Severe scoliosis (%)	17 (40)	25 (60)	42 (100)	24 (62)	15 (38)
Standing ability (%)	3 (5)	49 (95)	52 (100)	4 (11)	26 (89)
Truncal tone disorder (%)	27 (40)	40 (60)	67 (100)	13 (75)	5 (25)
Well-controlled epilepsy n. (%)	18 (35)	34 (65)	52 (100)	12 (38)	19 (62)
Intractable epilepsy	5 (15)	27 (85)	32 (100)	11 (100)	0 (0)
No epilepsy	6 (17)	29 (83)	35 (100)	5 (25)	15 (75)

**Table 2:** Contingency table comparing the cohorts of hospitals A and B.

		Pediatric Hospital A			Children Hospital B			Multicenter Total		
Independent Variables		Hip Dysplasia		*p* Value	Hip Dysplasia		*p* Value	OR	95% CIs	Z Statistic
		Yes	No		Yes	No				
Standing position	Yes	3	49	>0.0001	3	25	>0.0001	0.06	0.026–0.166	5.32
	No	31	37		25	9				
Independent walking	Yes	0	42	>0.0001	1	28	>0.0001	0.02	0.001–0.081	4.41
	No	37	41		26	7				
Spasticity	Yes	31	60	0.0038	28	1	>0.0001	29.01	6.67–124.14	4.61
	No	2	27		0	33				
MACS ≤ 4 vs. MACS 3	Yes	30	60	0.0038	24	3	>0.0001	8.42	3.37–21.04	4.47
	No	2	28		4	31				
Scoliosis	Yes	17	25	0.0311	24	15	0.0013	4.15	2.15–7.99	4.33
	No	16	62		4	19				
Truncal tone disorder	Yes	27	40	0.0004	13	5	0.0105	3.21	1.68–6.12	3.36
	No	6	47		15	29				
GMFCS ≤ 4 vs. GMFCS 3	Yes	35	57	0.0661	27	25	0.0172	4.03	1.58–10.24	3.10
	No	5	23		1	9				

**Table 3:** Multivariate logistic regression model.

Independent Variables	Z Test of Coefficients			
	Odds Ratio Estimate		Standard Error	Z Ratio
Intercept	−7.1046	0.0008	1.4281	−4.9749
Scoliosis (NS)	0.5887	1.8016	0.4940	1.1918
Truncal tone disorder (TT)	0.8882	2.4307	0.3160	2.8109
Spasticity (SP)	0.4762	1.6099	0.3067	1.5523
GMFCS score	0.3973	1.4878	0.38747	1.0256
MACS score	0.5217	1.6848	0.2811	1.8558
Epilepsy (E)	−0.5988	0.5494	0.3892	−1.5385
Etiology (ET)	0.2914	1.3382	0.3525	0.8267
Dystonia (D)	−0.3568	0.6999	0.5692	−0.6268
Sex (SE)	−0.5284	0.5895	0.4891	−1.0805
History of previous surgery (SU)	1.6501	5.2075	0.52796	3.1256

**Table 4:** Predictive performance metrics of ensemble classifiers (NN, SVM, LR) on the test set. Classifier-specific values for accuracy, sensitivity, and specificity, along with their 95% CI (confidence intervals) based on Wilson score intervals using confusion matrix data. Python library’s statsmodels were used for the calculation. AUROC confidence intervals were calculated using Python library’s scipy.stats for AUROC confidence intervals using the normal approximation.

Classifier	Accuracy	Accuracy CI (95%)	Specificity	Specificity CI (95%)	Sensitivity	Sensitivity CI (95%)	AUROC	AUROC CI (95%)
NN	0.84	[0.69, 0.92]	0.82	[0.64, 0.92]	0.89	[0.57, 0.98]	0.92	[0.83, 1.01]
SVM	0.81	[0.66, 0.91]	0.79	[0.60, 0.90]	0.89	[0.57, 0.98]	0.84	[0.72, 0.96]
LR	0.81	[0.66, 0.91]	0.82	[0.64, 0.92]	0.78	[0.45, 0.94]	0.8	[0.67, 0.93]

**Table 5:** Clinical Decision Support System input/output web interface screenshot.

Input of the patient data related to the risk of NHD will show as follows: 1. Neuromuscular scoliosis (NS); 2.Truncal tone (TT); 3. Spasticity (SP); 4. GMFCS(G): 5. 463 MACS(M); 6. Epilepsy (E); 7. Etiology (ET); 8. Dystonia (D); 9. Sex (SE); 10. Surgery (SU).						
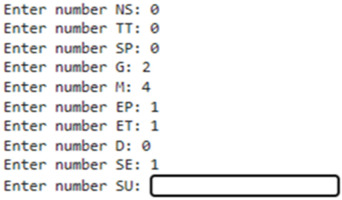						
Output (patient probability of having NHD) will show as follows: NN : probability of new patient of having NHD (NO–YES) : [0.96 0.04]; SVM : probability of new patient of having NHD (NO–YES) : [0.97 0.03]; LR : probability of new patient of having NHD (NO–YES) : [0.99 0.01]. Following Classifier-specific values for accuracy, sensitivity, and specificity, along with their 95% confidence intervals based on Wilson score intervals using confusion matrix data. Python libraries statsmodels were used for the calculation.						
**Classifier**	**Accuracy**	**Accuracy CI (95%)**	**Specificity**	**Specificity CI (95%)**	**Sensitivity**	**Sensitivity CI (95%)**
NN	0.84	[0.69, 0.92]	0.82	[0.64, 0.92]	0.89	[0.57, 0.98]
SVM	0.81	[0.66, 0.91]	0.79	[0.60, 0.90]	0.89	[0.57, 0.98]
LR	0.81	[0.66, 0.91]	0.82	[0.64, 0.92]	0.78	[0.45, 0.94]

**Table 6:** Comparison with previous metrics from literature sources.

First Author	Year	Subjects	Type of Predictors	AUC	Accuracy	Specificity	Sensitivity
Hermanson [7]	2015	145	Clinical and radiological	0.87	Not available	Not available	Not available
Bertoncelli [6]	2020	102	Clinical	Not available	77%	98%	43%
Pham [8]	2021	122	Radiological	Not available	91%	93%	88%
Bertoncelli—Current series	2025	182	Clinical	0.92	84%	89%	82%

## Data Availability

The original contributions presented in the study are included in the article, further inquiries can be directed to the corresponding author.
